# Assessing ChatGPT’s Reliability as an Educational Resource for Information on Breast Reduction Surgery

**DOI:** 10.7759/cureus.74698

**Published:** 2024-11-28

**Authors:** Emanuella M Brito, Isabel C Bernal, Andres A Mascaro-Pankova, James W Fletcher, Eric S Weiss, Christopher J Low, Michael C Cheung, Martin I Newman

**Affiliations:** 1 Surgery, Nova Southeastern University Dr. Kiran C. Patel College of Osteopathic Medicine, Fort Lauderdale, USA; 2 General Surgery, Hospital Corporation of America (HCA) Florida Westside Hospital, Plantation, USA; 3 Plastic and Reconstructive Surgery, Cleveland Clinic Florida, Weston Hospital, Weston, USA; 4 Plastic and Reconstructive Surgery, Broward Health Medical Center, Fort Lauderdale, USA; 5 Plastic and Reconstructive Surgery, Northeast Florida Plastic Surgery Center, Orange Park, USA; 6 Plastic Surgery, Cosmetic and Reconstructive Specialists of Florida PLLC, Fort Lauderdale, USA; 7 Plastic and Reconstructive Surgery, Cosmetic and Reconstructive Specialists of Florida PLLC, Fort Lauderdale, USA; 8 Plastic and Reconstructive Surgery, Cleveland Clinic Florida, Weston, USA

**Keywords:** artificial intelligence (ai), breast reduction surgery (brs), chatgpt, machine learning, patient education

## Abstract

Introduction: With advances in AI and machine learning, platforms like OpenAI’s ChatGPT are emerging as educational resources. While these platforms offer easy access and user-friendliness due to their personalized conversational responses, concerns about the accuracy and reliability of their information persist. As one of the most common surgical procedures performed by plastic surgeons worldwide, breast reduction surgery (BRS) offers relief for the physical and emotional burdens of large breasts. However, like any surgical procedure, it can raise a multitude of questions and anxiety.

Methods: To evaluate the quality of medical information provided by ChatGPT in response to common patient inquiries about breast reduction surgery, we developed a 15-question questionnaire with typical patient questions about BRS. These questions were presented to ChatGPT, and the answers were compiled and presented to five board-certified plastic surgeons. Each specialist categorized the response as (1) Appropriate, the response accurately reflects current medical knowledge and best practices for BRS; (2) No, not thorough, the response lacks sufficient detail to be a helpful educational resource; (3) No, inaccurate, the response contains misleading or incorrect information.

Results: A total of 75 survey responses were obtained, with five experts each analyzing 15 answers from ChatGPT. Of these, 69 (92%) responses were determined to be accurate. However, six (8%) responses were concerning to our experts: four (5.3%) lacked detail, and two (2.7%) were found to be inaccurate. Chi-square analysis revealed no statistical significance in the distribution of responses categorized as “accurate” versus “not thorough/inaccurate,” and “not thorough” versus “inaccurate” (p=0.778 and p=0.306, respectively).

Conclusion: While ChatGPT can provide patients with basic background knowledge on BRS and empower patients to ask more informed questions during consultations, it should not replace the consultation and expert guidance of a board-certified plastic surgeon.

## Introduction

Breast reduction surgery (BRS) offers significant relief for patients experiencing physical and emotional burdens due to large breasts and ranks as the 10th most common procedure performed by plastic surgeons globally [1]. However, the decision to undergo any surgical procedure often comes with a multitude of questions and anxieties. Potential complications, recovery timelines, and overall expectations are just a few of the patient’s more common concerns. To empower patients to make informed choices and optimize surgical outcomes, thorough patient education is paramount [2]. The internet has become a dominant source of health information, with patients increasingly turning to online resources to answer their medical queries before consulting a healthcare professional [3]. With the expansion of artificial intelligence (AI) and machine learning (ML), platforms like Open AI’s ChatGPT are threatening to replace “Dr Google'' as an educational resource. ChatGPT's ability to provide personalized answers in a conversational format is a significant draw for patients seeking information [4]. While these platforms offer undeniable advantages in terms of accessibility and user-friendliness, concerns linger regarding the accuracy and reliability of the information they provide [5].

This paper aims to evaluate the quality of medical information provided by ChatGPT in response to common patient inquiries about breast reduction surgery and further explore the potential of such platforms as a patient education tool for those considering this procedure.

## Materials and methods

Study design

We developed a 15-item questionnaire to encompass a wide range of topics commonly inquired about by patients considering breast reduction surgery (BRS). Questions are detailed in Table 1 and ChatGPT's responses are given in the Appendix. The questions were formulated based on their ability to address areas such as candidacy, surgical procedures, recovery, risks, and expected outcomes. The list of questions was also developed using resources from the American Society of Plastic Surgeons (ASPS) website, drawing from their compilation of frequently asked questions about BRS [6]. These questions were then used as prompts for OpenAI's ChatGPT version 3.5.0 (OpenAI, San Francisco, CA). Each question was presented to the language model independently, and its corresponding response was accessed on May 9, 2024. The full list of questions and their corresponding answers from ChatGPT is provided in the supplementary table. To assess the accuracy and suitability of the ChatGPT-generated responses for patient education, five board-certified plastic surgeons who specialize in BRS were recruited. A web-based survey platform, SurveyMonkey (SurveyMonkey Inc., San Mateo, CA), was utilized to present each ChatGPT response. The plastic surgeons were aware that the information they were evaluating AI-generated responses. Each evaluator anonymously and independently reviewed all responses and categorized them according to the following criteria: (1) Appropriate; the response accurately reflects current medical knowledge and best practices for BRS; (2) No, not thorough; the response lacks sufficient detail to be a helpful educational resource; (3) No, inaccurate; the response contains misleading or incorrect information. This categorization process aimed to systematically evaluate the quality of information provided by ChatGPT as a patient education tool for breast reduction surgery. To analyze the distribution of these expert ratings, we performed two independent chi-square tests.

**Table 1:** Common questions on BRS answered by ChatGPT Common breast reduction questions that were answered by ChatGPT. Discussing treatment, lifestyle, and recovery.

Question Number	Common Patient Questions About BRS
Q1	Who is a good candidate for breast reduction surgery (BRS)?
Q2	Who is not a good candidate for BRS?
Q3	What kind of surgeon performs BRS?
Q4	Does my surgeon need to be Board Certified in Plastic Surgery to perform a breast reduction surgery?
Q5	Does my surgeon need to have hospital privileges to perform a BRS?
Q6	Can I pick my “cup size” for a BRS?
Q7	Does breast reduction surgery leave scars?
Q8	Can I breastfeed following BRS?
Q9	Is breast reduction surgery painful?
Q10	Does breast reduction surgery include a breast lift?
Q11	How long is the recovery following breast reduction surgery?
Q12	Will breast reduction surgery alter my breast sensation?
Q13	Should I lose weight before getting breast reduction surgery?
Q14	Should I get breast reduction surgery before or after having kids?
Q15	What are the risks of breast reduction surgery?

Statistical analysis

To assess the adequacy of ChatGPT’s responses, we employed chi-square tests using IBM SPSS Statistics 28.0 (IBM Corp., Armonk, NY). We conducted two independent chi-square analyses. The first analysis examined the relationship between individual survey responses and a binary categorization of answer appropriateness to each question as “sufficient” (yes) or “not thorough or inaccurate” (no). The second analysis investigated the statistical significance between the responses categorized as “no, not thorough” and those categorized as “no, inaccurate.” Statistical significance was assessed using a p-value threshold of 0.05.

## Results

Our survey of five board-certified plastic surgeons who routinely perform BRS noted some issues with the ChatGPT responses. Of the five plastic surgeons, three had > 10 years of experience and all five noted to be board-certified in plastic surgery. Our response rate was 100%. 

**Figure 1:**
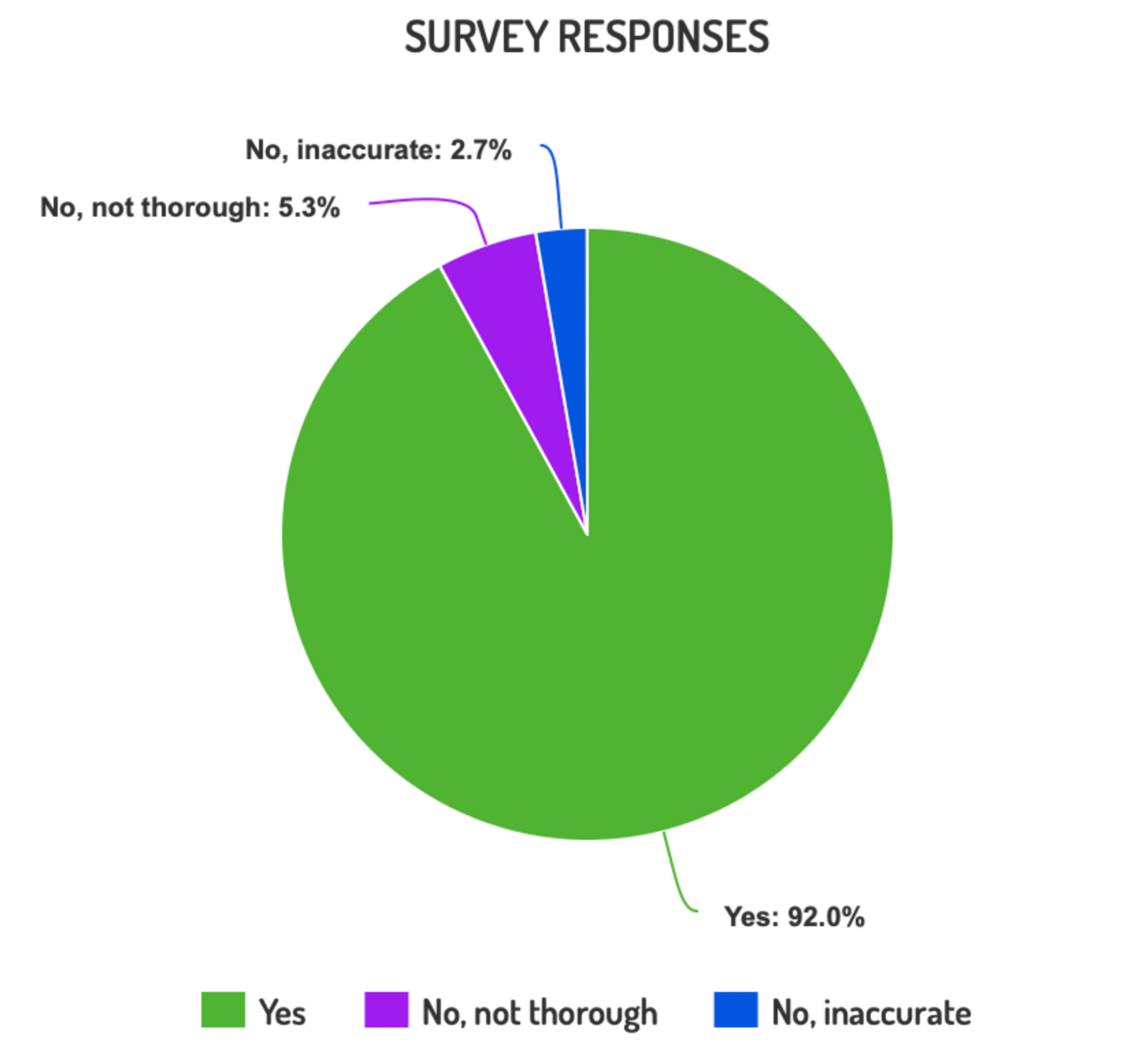
Survey Results: ChatGPT Answer Analysis Pie chart of survey responses for ChatGPT answer analysis by five board-certified plastic surgeons. Total of 75 survey responses in which 92% (blue) were classified as accurate; 5% (green) were classified as not thorough; and 3% (purple) as inaccurate.

Our five experts each analyzed 15 AI-generated responses; a total of 75 responses were then statistically analyzed by independent chi-square tests. Of these 75 ratings by our experts, 69 (92.0%) were in agreement as appropriate responses. However, six (8.0%) separate and distinct AI-generated responses gave concern to our experts as follows. Four (5.3%) ChatGPT responses were noted to lack detail, and two (2.7%) were found to be inaccurate (Figure 1). There was no overlap in this respect. In no case did more than one expert find an issue with any particular response. While these findings suggest ChatGPT's potential as a tool for general information on breast reduction surgery, it may not be sufficient for addressing in-depth or nuanced questions. Figure 1 explains the response breakdown.

The first chi-square test results are presented in Table 2. A chi-square test of independence was performed to evaluate the relationship between the responses categorized as “sufficient” (yes) versus “not thorough/inaccurate” (no). The relationship between these variables was not statistically significant (p<0.05), X^2^, (14, N=75) = 9.783, p = .778. However, this does not necessarily mean all ChatGPT responses were acceptable.

**Table 2:** Chi-Square Test of ChatGPT for Answer Appropriateness Chi-square analysis evaluating the relationship between individual survey responses “sufficient” vs “Not thorough/Inaccurate." ^a^30 cells (100.0%) have expected count less than 5. The minimum expected count is .40.

	Value of X^2^	df	p-value (two-sided)
Pearson chi-square	9.783^a^	14	0.778
Likelihood ratio	11.791	14	0.623
No. of survey responses	75		

A chi-square test was performed to evaluate the relationship between the responses categorized as “not thorough” versus “inaccurate”, shown in Table 3. The relationship between these variables was not statistically significant (p<0.05), X^2^, (5, N=6) = 6, p = .306. 

**Table 3:** Chi-Square Test of ChatGPT for Not Appropriate Answers Chi-square analysis evaluating the relationship between individual survey responses “Not thorough" vs "Inaccurate." ^a^12 cells (100.0%) have expected count less than 5. The minimum expected count is .33.

	Value of X^2^	df	p-value (two-sided)
Pearson chi-square	6.000^a^	5	0.306
Likelihood ratio	7.638	5	0.177
No. of survey responses	6		

Although these discrepancies did not reach statistical significance, it is still notable that six out of 75 ratings (8%) were classified as inaccurate, as any form of misinformation can have clinical implications. Therefore, we will examine and discuss the answers to the questions where at least one respondent categorized a ChatGPT answer as insufficient or inaccurate. This may provide insight into the type of information ChatGPT’s responses might lack.

The four questions that at least one expert deemed lacking sufficient detail were questions 1, 2, 6, and 10, while questions 7 and 8 were deemed inaccurate by at least one expert (Table 1).

## Discussion

The increasing tendency of patients to turn to large language models like ChatGPT along with the increased availability of this common internet tool raises concerns about the accuracy of medical information patients receive. This study aimed to evaluate the accuracy of information ChatGPT provides to patients considering a commonly performed plastic surgical procedure, in this case, breast reduction surgery. Our analysis as outlined above revealed that six (40%) of the 15 AI-generated responses were in some manner in conflict with the opinions of professional board-certified plastic surgeons. While these discrepancies did not achieve statistical significance, six (8%) out of 75 ratings classified as inaccurate information is still noteworthy, as any kind of misinformation can be clinically significant. Sensitive areas such as surgery must heavily rely on evidence-based medicine, as misleading data in such fields can have real-world implications capable of affecting patients' lives. Especially in plastic surgery, where most cases of breast reduction procedures are elective, risks should be minimized in all aspects, starting with a patient’s understanding of the procedure. A misinformed patient can lead to unrealistic or unreasonable expectations and, ultimately, a decrease in patient satisfaction and patient-reported outcomes. 

As a growing field, discussing the accuracy of information provided by artificial intelligence is crucial for ensuring clarity for individuals using these platforms. AI developers must be aware of the implications that misinformation can have on patients and use this awareness as a motive to ensure the continuous improvement of information provided by AI. Patients may not always be able to distinguish between accurate and misleading information. Therefore, understanding that machine learning programs are subject to errors, any medical advice provided by such platforms should be accompanied by a disclaimer that it does not substitute for professional medical advice. Transparency is key to implementing ethical parameters and guidelines in this ever-changing field of technology and artificial intelligence. It is essential to make it clear to users that any information provided should be confirmed by an accredited professional. In this paper, ChatGPT largely emphasized the importance of discussing questions with a board-certified plastic surgeon. However, it did not include statements about the possibility of inaccurate or incomplete information.

Four of the six questions that conflicted with the experts’ opinions were classified as lacking sufficient detail. These were questions 1, 2, 6, and 10 (Table 1). In response to question 1, which inquired, 'Who is a good candidate for breast reduction surgery?' ChatGPT elaborates on the characteristics that patients must have to be considered good candidates. These include physical discomfort, skin issues, postural problems, emotional distress, a clear understanding of the procedure’s risks and outcomes, overall health, non-smoking status, and psychological readiness. All of these factors are also classified by the ASPS as important criteria to determine a patient’s eligibility for BRS. However, ChatGPT fails to include two factors highlighted by ASPS as good indicators for undergoing a BRS: shoulder indentations caused by bra straps and non-vaping status within the broader category of non-smokers [7].

ChatGPT answers question 2, 'Who is not a good candidate for BRS?' by listing the characteristics of a not-ideal candidate for breast reduction surgery. These include patients with serious comorbidities, smokers, patients with unrealistic expectations, or those planning to undergo significant weight loss. It also addresses those who are currently pregnant or breastfeeding by stating: “Women who are currently pregnant or breastfeeding are advised to wait until after they have finished nursing. Pregnancy and breastfeeding can alter breast size and shape, which would affect the surgical outcome.” However, it makes no mention of someone who is “actively trying” to become pregnant or someone who may be interested in getting pregnant within the foreseeable future. Because pregnancy increases breast hypertrophy, and because breast surgery has unpredictable effects on the ability to breastfeed, a more inclusive response from the AI might also include those with plans to become pregnant in the immediate future [8,9]. A current study has shown a reduction in the breastfeeding success rate by 41% following breast reduction surgery [10]. Such a high decline in breastfeeding success following breast surgery reinforces the need for patients who seek to become pregnant after the surgery to be aware of this possible complication. 

Question 6 addressed whether patients can choose their own 'cup size' for breast reduction surgery (BRS). The AI platform explains that this decision depends on a discussion between the patient and the surgeon. An underlying theme of this response implies a significant latitude on the part of the operating surgeon to tailor the results of this procedure to a patient’s expectations and desires. Although some latitude is possible when performing breast reduction surgery, the outcomes are more closely tied to the presenting size and shape of the preoperative breast and body habitus. Discussing “the look and feel you are aiming for” is important, but the response could be more complete if it included a further discussion of what “is” and what “is not” possible to achieve safely. In addition, the use of “sizers during the consultation to help you visualize potential outcomes” has more of a role when discussing breast augmentation surgery and not breast reduction surgery [11].

While ChatGPT effectively explains the mechanics of breast lift procedures and their relation to breast reduction surgery, it fails to address the crucial factor of individual patient assessment in its response to question 10, which asked if breast reduction surgery includes a breast lift. Unlike a one-size-fits-all approach, each patient is carefully evaluated for their suitability for either a breast lift or reduction. ChatGPT's description of the breast lift's purpose aligns with the ASPS website, stating the primary goal as lifting and reshaping sagging breasts. This is achieved by removing excess skin and tightening surrounding tissues to create a new, supported breast contour [6]. However, the AI platform does not explain the technical differences between both procedures. Although it is possible and common for them to be done together, not all good candidates for breast reduction are also good candidates for mastopexy or breast lift. Mastopexy will have a greater impact on the nipple and areola positioning, and patients usually seeking this procedure will have greater aesthetic concerns [12]. Therefore, a personalized and tailored approach should be the aim of surgeons assessing patients seeking BRS to determine the indication of a combined procedure with a breast lift.

Of the six questions classified as inaccurate, questions 7 and 8 were deemed incorrect by at least one expert (Table 1). In response to question 7, which asked whether BRS leaves scars, ChatGPT confirms that BRS does indeed leave scars. It explains that the pattern of scars depends on the type of incision made by the surgeon, which can be one of three types: anchor or inverted-T incision, vertical or “lollipop” incision, and periareolar or “donut” incision. The OpenAI platform emphasizes the need for patients to discuss with their plastic surgeons the location of their scars and what to expect during the healing process. This allows patients to have realistic expectations about their surgical outcomes. However, the platform attributes the main cause of scar formation to the surgical technique and to the degree of breast reduction. Although different techniques and the extent of reduction do affect scar formation, they are not the only factors. Skin loss and delayed wound healing have been reported as the number one complication encountered in reduction mammoplasty [13]. ChatGPT fails to mention the probability of such complications occurring. Scarring is a complex process, not solely dependent on a surgeon’s technique. Wound healing is affected by individual characteristics, including genetics, ethnicity, age, and history of hypertrophic scars. These are also known as non-modifiable circumstances that must be explained to the patient by their surgeon so both can work together to optimize the healing process [14]. 

Lastly, regarding question 8 about breastfeeding after breast reduction surgery, ChatGPT notes that while breastfeeding is possible, success is not guaranteed and depends on factors such as the surgical technique and the extent of tissue removed. The platform briefly explains different factors affecting breastfeeding after BRS, such as surgical technique, amount of tissue removed, preservation of nipple sensation, and individual variability. When discussing surgical techniques that allow for a greater chance of breastfeeding, ChatGPT references the “pedicle method,” stating that it has the best outcome for preserving breastfeeding capability. However, this is an umbrella term for different techniques involving multiple pedicles in the breast, all aiming to maintain nerve and blood supply to the nipple [15]. While the AI response offers broad information relevant to a plastic surgeon consultation, it overlooks a crucial point. It's important to understand that difficulty breastfeeding is common, affecting roughly 70% of women regardless of breast reduction surgery [16]. Studies show a 62% success rate for breastfeeding after reduction mammaplasty [17]. This can be compared to the national average of approximately 41.8% of women who ultimately discontinue breastfeeding altogether after six months post-partum [18]. This broader perspective, which ChatGPT missed, is vital for patients considering reduction surgery. While the AI acknowledges potential breastfeeding challenges, emphasizing the overall picture is essential for informed decision-making.

It is important to acknowledge the potential benefits of patients using AI for introductory surgical queries. Information provided by AI sources like ChatGPT can empower patients to ask more informed questions during consultations with their board-certified plastic surgeons. This can facilitate a deeper understanding of potential risks, benefits, and alternatives associated with BRS, ultimately leading to a more informed consent process.

Limitations

Although this study has successfully demonstrated the potential use of ChatGPT in providing useful preliminary information for patients seeking breast reduction surgery, it had some limitations. A larger sample size, both in the number of questions and specialists, could change the chi-square results and consequently the significance of the answers provided by ChatGPT. Additionally, AI is a trainable system that is capable of learning and drawing new conclusions as it comes across more data [19]. Therefore, the quality of information provided by ChatGPT for this study could improve in a very short period of time. Consequently, the data obtained by this study should be viewed as a current snapshot of ChatGPT’s information on BRS, which may change in the future. Another important limitation is ChatGPT's limited ability to address individual cases. Due to the absence of knowing patient-specific medical history and surgeon expertise, ChatGPT is limited in its ability to provide personalized answers to medical questions.

## Conclusions

AI platforms such as ChatGPT are emerging as potential educational tools in healthcare offering patients easy access to information in a conversational format. This study investigated the quality of information ChatGPT provides for BRS patients, evaluating its suitability as a reliable information source, and found a notable rate of inaccuracies and inconsistencies generated by this AI tool. Surgeons and patients should be aware of these inaccuracies and inconsistencies to avoid any potential complications caused by a misinformed patient. AI generators should use the real-world impact of healthcare misinformation on patients’ lives as a motive to seek continuous improvement based on current evidence-based medicine. Additionally, transparency should be a priority, with disclaimers added to every machine learning platform when providing healthcare information. Any information from a machine learning platform, while often instructive, should always be confirmed by an accredited physician. Therefore, while ChatGPT can provide patients with basic background knowledge on BRS and empower patients to ask more informed questions during consultations, it should not replace the consultation and expert guidance of a board-certified plastic surgeons. 

## Appendices

**Table 4:** ChatGPT answers for BRS related questions

Questions	ChatGPT answers
Q1. Who is a good candidate for Breast Reduction surgery (BRS)?	Breast reduction surgery, also known as reduction mammaplasty, is a procedure designed to reduce the size and weight of large breasts. Individuals may consider this surgery for various reasons, including physical, emotional, and aesthetic concerns. Good candidates for breast reduction surgery typically include those who: Experience Physical Discomfort: Women or men with disproportionately large breasts often suffer from physical discomfort such as back, neck, and shoulder pain. The weight of excess breast tissue can impair the ability to lead an active life and participate in physical activities. Have Skin Issues: Large breasts can lead to skin irritation beneath the breast fold, including rashes and skin infections. Suffer from Postural Problems: The excess weight of large breasts can affect posture, leading to secondary problems like chronic pain in the spine and surrounding muscles. Are Self-Conscious or Experience Emotional Distress: Emotional distress due to unwanted attention, difficulty in finding appropriate clothing, or self-image issues related to large breasts are common motivations for seeking breast reduction. Have Realistic Expectations: Good candidates understand the potential benefits and limitations of the surgery, including the fact that scarring is inevitable, although it can often be placed in less visible locations. Are in Good General Health: As with any surgery, candidates should be in good health, which helps reduce the risk of complications during and after surgery. This includes being at a stable weight, as significant weight fluctuations can affect surgical outcomes. Non-smokers: Smoking can impair healing and increase the risk of complications during and after surgery. Patients are generally advised to quit smoking well in advance of the procedure. Understand the Risks: Like all surgeries, breast reduction involves certain risks such as bleeding, infection, and adverse reactions to anesthesia. Candidates should be aware of these risks and discuss them thoroughly with their surgeon. Psychological Readiness: The candidate should be psychologically stable and have a strong personal motivation for undergoing the procedure that is not influenced by external pressures. The first step for anyone considering breast reduction surgery is to consult with a qualified plastic surgeon. During this consultation, the surgeon will evaluate the individual’s overall health, discuss expectations and desired outcomes, and explain the potential risks and benefits of the procedure. This helps ensure that the candidate's goals align with what can realistically be achieved through surgery.
Q2. Who is not a good candidate for BRS?	Certain individuals may not be ideal candidates for breast reduction surgery (BRS) due to various health, lifestyle, or psychological factors. Here are some examples of who might not be suitable for this procedure: Those with Serious Health Issues: Individuals with major health conditions that could complicate surgery or recovery, such as heart disease, severe diabetes, or blood clotting disorders, may not be good candidates. These conditions can increase the risk of surgical complications. Smokers: Smoking can significantly impair healing and increases the risk of complications during and after surgery. Patients who are unwilling or unable to quit smoking may be advised against undergoing breast reduction surgery. Those with Unrealistic Expectations: Individuals expecting perfection or hoping for a dramatic alteration in body image might be disappointed. It's important for candidates to have realistic expectations about the results and understand the potential scars and recovery process. Individuals who are Pregnant or Breastfeeding: Women who are currently pregnant or breastfeeding are advised to wait until after they have finished nursing. Pregnancy and breastfeeding can alter breast size and shape, which would affect the surgical outcome. Those Planning Significant Weight Loss: Individuals who intend to lose a significant amount of weight might consider delaying the surgery. Weight loss can naturally reduce breast size and might change the desired outcome of the surgery. Very Young Patients: Young women whose breasts have not fully developed are typically advised to wait until their breasts have matured before considering breast reduction surgery. Individuals with Active Infections or Poor Wound Healing: Conditions that impair wound healing, such as chronic skin infections or systemic diseases like lupus, might disqualify someone from undergoing surgery until those issues are managed. Those Under Severe Psychological Distress or External Pressure: Individuals who are not psychologically stable or are considering the surgery mainly due to pressure from others may need to address these issues before undergoing any elective surgical procedure. For anyone considering breast reduction surgery, a detailed consultation with a qualified plastic surgeon is essential. This consultation should thoroughly evaluate the individual’s health, discuss their motivations and expectations, and consider their overall readiness for surgery. This helps ensure that the decision to proceed with breast reduction is made in the best interests of the patient's health and well-being.
Q3. What kind of surgeon performs BRS?	Breast reduction surgery (BRS) is typically performed by a plastic surgeon, specifically one who specializes in aesthetic and reconstructive surgeries. Plastic surgeons are extensively trained to perform surgeries that modify or reconstruct parts of the body, with the goal of improving function and appearance. Here's a breakdown of the qualifications and training typically required of a plastic surgeon who performs breast reduction surgery: Medical Degree: Like all physicians, plastic surgeons begin their training by earning a medical degree (MD or DO) from an accredited medical school. Residency: After completing medical school, prospective plastic surgeons undergo a residency program in plastic surgery. This is an intensive period of training lasting several years, where they gain comprehensive experience in all aspects of plastic surgery, including both cosmetic and reconstructive procedures. Some may first complete a residency in general surgery followed by a specialized residency in plastic surgery. Board Certification: In the United States, plastic surgeons are certified by the American Board of Plastic Surgery (ABPS) or, in some cases, the American Osteopathic Board of Surgery. Board certification involves passing rigorous oral and written exams, as well as demonstrating specific competencies in the field of plastic surgery. Fellowships and Continuing Education: Many plastic surgeons also choose to complete additional fellowships in sub-specialties of plastic surgery, such as breast surgery, to further refine their expertise. Continuing medical education is required to maintain board certification and stay updated with the latest advancements in plastic surgery techniques and safety protocols. State Licensure: Like all physicians, plastic surgeons must be licensed in the state where they practice. This requires passing a series of examinations and maintaining an ethical standard of practice. When considering breast reduction surgery, it's important for patients to seek a surgeon who has significant experience with the procedure. It's also helpful to review the surgeon's before and after photos of previous surgeries, check their credentials, and read reviews from other patients. Initial consultations are crucial for understanding the surgeon's approach and for discussing expectations, potential outcomes, and risks associated with the surgery. This helps in making an informed decision and establishing a comfortable rapport with the surgeon.
Q4. Does my surgeon need to be Board Certified in Plastic Surgery to perform a BRS?	Yes, it is highly advisable that your surgeon be board certified in plastic surgery to perform a breast reduction surgery (BRS). Board certification in plastic surgery ensures that the surgeon has undergone rigorous training specifically in the field of plastic surgery and has met the stringent standards set by the certifying board. This certification provides a measure of assurance regarding the surgeon's qualifications and expertise in performing complex surgical procedures, including breast reduction. Here are a few reasons why choosing a board-certified plastic surgeon is important: Specialized Training: Board-certified plastic surgeons have completed extensive training in plastic surgery, including years of residency that focus solely on plastic and reconstructive procedures. This training is crucial for the complex anatomical and aesthetic considerations involved in breast reduction surgery. Adherence to High Standards: Board certification requires surgeons to adhere to high ethical and professional standards. Certified surgeons must also participate in continuing education, which helps them stay updated on the latest surgical techniques and safety protocols. Peer Recognition: To become board certified, surgeons must pass rigorous examinations and often need to present cases they have handled to a panel of peers. This peer review process helps ensure that the surgeon’s skills and knowledge meet the high standards of the plastic surgery profession. Patient Safety and Trust: Choosing a board-certified plastic surgeon helps ensure that your procedure will be performed safely and with the highest standards of care. It also provides peace of mind knowing that your surgeon has a verified background of training and expertise. While technically any licensed medical doctor can perform surgeries within the scope of their professional licenses, having a board-certified plastic surgeon perform your breast reduction is crucial for ensuring the best outcomes and minimizing risks. Always verify your surgeon’s credentials and ask about their specific experience with breast reduction surgeries during your initial consultation.
Q5. Does my surgeon need to have hospital privileges to perform a BRS?	Yes, it is generally important for your surgeon to have hospital privileges to perform breast reduction surgery (BRS). Hospital privileges refer to the authorization granted by a hospital to a physician to perform specific procedures at that hospital. This is an important aspect of ensuring patient safety and high standards of care. Here are several reasons why hospital privileges are crucial: Peer Review and Credentialing: Hospital privileges are granted after a thorough credentialing process that includes verifying the surgeon's education, training, experience, and competence. This peer review ensures that only qualified surgeons are allowed to perform surgeries in the hospital. Safety and Emergency Preparedness: Having hospital privileges means that the procedure is conducted in a facility that is equipped to handle emergencies should they arise. Hospitals have the necessary staff, equipment, and protocols to manage unexpected complications during or after surgery. Standard of Care: Hospitals maintain strict standards regarding patient care. Surgeons with hospital privileges are required to adhere to these standards, which includes following best practices and maintaining a commitment to patient safety. Continuity of Care: If there are post-operative complications, surgeons with hospital privileges can admit patients to the hospital where they operate. This ensures continuity of care and access to all necessary medical resources. Surgeon’s Reputation and Professional Standing: Typically, hospitals only grant privileges to surgeons who are in good standing in their professional community. This serves as an additional level of reassurance about the surgeon’s qualifications and ethical conduct. While many breast reduction surgeries can be performed safely in accredited outpatient surgical centers, having a surgeon with hospital privileges is a key indicator of their competence and credibility. Even if the surgery is planned for an outpatient setting, knowing that the surgeon has the capability to transfer and treat you in a hospital if necessary is important for your overall safety and peace of mind. When consulting with potential surgeons for your breast reduction surgery, it's a good idea to ask about their hospital privileges, including where they have the ability to treat patients if hospitalization becomes necessary.
Q6. Can I pick my “cup size” for a BRS?	Choosing a specific "cup size" for breast reduction surgery (BRS) isn't as straightforward as it might seem. Unlike shopping for a bra, where you can select a desired cup size, surgical adjustments during breast reduction focus more on achieving a breast size that is proportionate to your body frame, alleviates discomfort, and meets your aesthetic goals. Here are some key points to consider: Communication of Goals: During consultations, it's important to communicate your expectations and desired outcomes with your surgeon. Discussing your lifestyle, aesthetic preferences, and the physical issues you're experiencing due to your current breast size helps the surgeon understand your goals. Variability in Cup Sizes: Bra cup sizes are not standardized and can vary significantly between different lingerie brands and styles. Therefore, mentioning a specific cup size can be ambiguous and may not accurately convey your desired outcome. Assessment by the Surgeon: Your surgeon will assess your body structure, including your chest width, existing breast tissue, and skin elasticity, to recommend a breast size that is proportionate and balanced for your body. They will consider factors like how much tissue needs to be removed to alleviate any symptoms (such as back pain) you might be experiencing. Realistic Expectations: Surgeons can give you an approximate idea of how your breasts will look post-surgery rather than guaranteeing a specific cup size. They often use before-and-after photos of previous patients with a similar physique and desired outcome to help set realistic expectations. Simulation and Sizers: Some plastic surgeons offer 3D imaging or use sizers during the consultation to help you visualize potential outcomes. This can be a helpful tool in understanding how different sizes might look on your body. Focus on Appearance and Comfort: Ultimately, the goal of breast reduction is to achieve breasts that are in better proportion to your body and relieve any discomfort caused by overly large breasts. The emphasis is typically on creating a natural look and improving quality of life rather than achieving a specific cup size. When preparing for your consultation, it might be more effective to discuss the look and feel you are aiming for, rather than focusing solely on achieving a certain cup size. This approach allows your surgeon to tailor the procedure to best meet your physical and aesthetic needs.
Q7. Does BRS leave scars?	Yes, breast reduction surgery (BRS) does leave scars. However, plastic surgeons are trained to place these scars in areas that are less noticeable and use techniques that aim to minimize scar visibility. The pattern and extent of the scars depend on the surgical technique used and the amount of reduction needed. Here are the most common types of incisions and resulting scars from breast reduction surgery: Anchor Incision (Inverted-T Incision): This is the most common technique for those requiring significant reductions. The incision circles around the areola, goes vertically down to the breast crease, and then horizontally along the breast crease in a line that resembles an anchor or an inverted "T". This method allows for substantial reshaping and size reduction but results in the most noticeable scarring. Vertical or “Lollipop” Incision: This method involves an incision around the areola and a vertical incision down to the breast crease, resembling a lollipop. It is suitable for moderate reductions and results in less scarring than the anchor technique. Periareolar or “Donut” Incision: This technique involves an incision solely around the areola. It is generally used for smaller reductions and adjustments, resulting in minimal scarring. However, it allows for less dramatic reshaping than the other methods. Scar Management and Healing: Initial Appearance: Scars are typically most noticeable right after surgery. They may be red, raised, and visible. Maturation Process: Over time, usually within a year, scars typically fade and flatten. The final appearance of scars can depend on factors like genetics, skin color, and how well the post-operative care guidelines are followed. Post-Operative Care: Surgeons often recommend various treatments to aid scar healing and improve their appearance. These might include silicone sheets or gels, steroid injections, laser treatments, and proper sun protection. Individual Variation: Healing and scar visibility can vary widely between individuals. Some people may heal with minimal scarring, while others may develop more pronounced scars. It's important to discuss scarring with your plastic surgeon during the consultation. Understanding where the scars will be located, what they will look like, and how to care for them post-surgery can help set realistic expectations and contribute to a more satisfactory outcome.
Q8. Can I breast feed following BRS?	Breastfeeding after breast reduction surgery (BRS) is possible, but it can be more challenging and is not guaranteed. The likelihood of being able to breastfeed depends on several factors related to the surgical technique used and the extent of the tissue removed. Here are some key considerations: Surgical Technique: The ability to breastfeed post-surgery largely depends on how the surgery was performed. Techniques that preserve the connections between the nerves of the nipple and the milk ducts enhance the chances of successful breastfeeding. For example, the pedicle method, where the nipple is left attached to a stalk of tissue that preserves its blood and nerve supply, tends to have better outcomes for preserving breastfeeding capability compared to techniques where the nipple is completely detached and then reattached as a free graft. Amount of Tissue Removed: The more breast tissue that is removed, the fewer milk-producing glands are left intact, which can reduce the amount of milk a woman is able to produce. Nipple Sensation: Preservation of nipple sensation is a good indicator of intact nerve connections, which are important for milk production and ejection. Loss of sensation can suggest that breastfeeding might be more difficult. Individual Variability: Each person heals differently, and the individual's ability to produce enough milk can vary widely even without surgery. Preparing to Breastfeed after BRS: Consultation Before Surgery: Discuss your desire to breastfeed with your surgeon before the procedure. This can influence the surgical technique used and increase the likelihood of preserving breastfeeding function. Post-Surgery Lactation Consultation: Working with a lactation consultant can be beneficial. They can provide strategies and support to help stimulate milk production and address any breastfeeding challenges. Supplemental Feeding: Be prepared for the possibility that you might not be able to exclusively breastfeed. Many mothers find they need to supplement with formula or donated breast milk. Many women have successfully breastfed after breast reduction surgery, though they may face challenges such as reduced milk supply. It’s important to set realistic expectations and prepare for the need to supplement breastfeeding if necessary. If breastfeeding is a significant concern for you, be sure to discuss it thoroughly with your surgeon and a lactation consultant to get personalized advice and support.
Q9. Is BRS painful?	Yes, as with any surgical procedure, breast reduction surgery (BRS) can be associated with some level of pain and discomfort during the recovery period. However, the extent of pain experienced can vary widely among individuals and depends on several factors such as the surgical technique used, the individual's pain tolerance, and the overall complexity of the surgery. Postoperative Pain Management: Immediately After Surgery: Immediately following the surgery, patients are typically given pain medication to manage discomfort. This is often administered intravenously or through pain pumps. First Few Days: In the days following the procedure, you are likely to experience soreness, swelling, and bruising, which can be managed with prescribed oral pain medications. Following Weeks: As the healing process continues, the intensity of pain and discomfort should gradually decrease. Over-the-counter pain relievers like acetaminophen or ibuprofen are often sufficient to manage any lingering discomfort during this period. Other Common Sensations: Tightness: Many patients report feeling tightness or a sensation of pressure around the chest area as the skin and tissues adjust to the breasts' new size. Numbness or Tingling: Temporary numbness or tingling around the surgical sites or in the nipples can occur due to nerve disturbance during surgery. This usually resolves on its own but may take several months. Pain Duration and Management: Duration: The most significant discomfort typically occurs within the first week post-surgery, and significant improvements often happen after the first few weeks. Management: Effective pain management is crucial and can include medications, ice packs, rest, and careful activity management. Your surgeon will provide detailed instructions on how to manage postoperative pain, including guidelines on activity restrictions to aid in recovery. Long-Term Recovery: Follow-Up Appointments: Regular follow-ups with your surgeon will help ensure that your recovery is progressing well and that any pain or complications are addressed promptly. Emotional Support: It’s also important to consider emotional support, as recovery from surgery can be both physically and emotionally taxing. Overall, while breast reduction surgery involves some level of postoperative pain, many patients find the long-term benefits of relief from symptoms like back and neck pain, skin irritation, and discomfort from overly large breasts to outweigh the temporary discomfort from the surgery. Pain management strategies and close communication with your surgical team are key to a smoother recovery.
Q10. Does BRS include a breast lift?	Yes, breast reduction surgery (BRS) typically includes elements of a breast lift (mastopexy). The procedures are often combined because the goal is not only to reduce the size of the breasts but also to improve their shape and position on the chest. Here's how these procedures relate and function together: Breast Reduction Surgery Purpose: The primary goal of breast reduction is to remove excess breast tissue, fat, and skin to achieve a breast size in proportion with your body and to alleviate discomfort caused by overly large breasts. Technique: This involves various incision patterns and techniques, depending on the amount of reduction needed, the existing breast anatomy, and patient-surgeon preferences. Common techniques include the anchor incision, vertical (or "lollipop") incision, and periareolar incision. Breast Lift (Mastopexy) Purpose: The primary objective of a breast lift is to raise and reshape sagging breasts. This is achieved by removing excess skin and tightening the surrounding tissue to support the new breast contour. Technique: The incision patterns for a breast lift can be similar to those used in breast reduction, especially the anchor and lollipop techniques, depending on the degree of lift required. Combining the Procedures Aesthetic and Functional Improvement: Both procedures aim to improve the breasts' aesthetic appearance and functional issues. When performing a breast reduction, the surgeon typically repositions the nipple and areola to a higher, more youthful position, which is a key component of a breast lift. Scarring and Technique: The scarring for both procedures can be similar since they may use the same incision patterns. The choice of pattern depends on the amount of lifting and reshaping needed alongside the reduction. Outcome: The combined outcome leads to smaller, lighter, and aesthetically pleasing breasts that are well-proportioned to the rest of the body and positioned higher on the chest. During a consultation for breast reduction, it's important to discuss with your surgeon how they will address both the size and the position of the breasts. Most patients undergoing breast reduction are also looking to achieve a more lifted and youthful breast contour, which a combined reduction and lift procedure can effectively accomplish.
Q11. How long is the recovery following BRS?	Recovery from breast reduction surgery (BRS) can vary depending on several factors, including the extent of the surgery, the individual's general health, the technique used, and how quickly your body heals. However, there is a general timeline you can expect for recovery: Initial Recovery Phase First Few Days: Immediately after the surgery, you will likely experience pain, swelling, and soreness. Pain medication will be prescribed to manage discomfort. Most patients are advised to wear a support bra or a compression garment to help reduce swelling and support the healing breasts. First Week: You should rest and limit physical activities. It's crucial to avoid any heavy lifting or strenuous activities that could strain the incisions or affect healing. Many patients can return to work within 1-2 weeks, depending on the nature of their job (office work may be resumed sooner than physically demanding jobs). Ongoing Recovery First Month: Swelling and bruising should gradually decrease during the first month. You can slowly start to resume more activities as comfort permits. However, high-impact activities should still be avoided. Six Weeks: Most normal activities, including some forms of exercise, can typically be resumed around this time. Your surgeon will give you specific guidelines based on your progress. Long-Term Healing Three to Six Months: By this time, most patients feel back to normal and can see the more settled appearance of the surgical outcome. However, minor swelling may persist for up to a year. One Year: Scars will continue to mature and fade over this period. It can take a year or longer for scars to flatten and lighten in color. Tips for a Smooth Recovery Follow Post-Operative Instructions: Adhering closely to your surgeon's instructions can help speed up your recovery, reduce the risk of complications, and improve the cosmetic outcome. Attend Follow-up Appointments: These are essential to ensure your incisions are healing properly and to monitor your overall recovery. Any concerns about healing and results can be addressed during these visits. Maintain a Healthy Lifestyle: Eating a balanced diet, staying hydrated, and avoiding smoking can significantly enhance your recovery process. It's important to have realistic expectations and understand that full recovery and seeing the final results will take time. Every individual heals at a different rate, and your surgeon is your best resource for questions about what you can expect during your specific recovery process.
Q12. Will BRS alter my breast sensation?	Breast reduction surgery (BRS) can affect breast sensation, particularly in the nipples. Changes in sensation are due to the surgical alteration of breast tissue and the potential impact on nerves during the procedure. Here are some key points regarding how breast reduction surgery might affect sensation: Potential Changes in Sensation Temporary Numbness or Increased Sensitivity: It's common for patients to experience temporary numbness or increased sensitivity in their nipples and breast skin after surgery. This typically results from nerves being stretched, compressed, or severed during the procedure. Permanent Changes: While many patients recover sensation over time, some may experience permanent changes in nipple or breast sensation. The extent and permanence of these changes can depend on the surgical technique used and how individual bodies heal. Factors Influencing Sensory Changes Surgical Technique: Techniques that involve moving the nipple (such as the pedicle method where the nipple is left attached to a stalk of tissue) generally preserve more nerve connections compared to methods where the nipple is removed and reattached as a free graft. Amount of Tissue Removed: Larger reductions might involve more extensive tissue and nerve disruption, potentially leading to a higher risk of altered sensation. Individual Healing: Healing varies widely among individuals. Some patients may regain full sensation, while others might not. Recovery and Regaining Sensation Time Frame: Sensation that is affected by surgery can sometimes take up to a year or more to stabilize. During this period, the nerves may gradually recover and regenerate, potentially restoring normal or near-normal sensation. Progress: The recovery of sensation can be gradual and may not be entirely predictable. Patients often report improvements over many months following the procedure. Discussing Concerns with Your Surgeon It’s important to discuss the potential for changes in sensation with your surgeon during the consultation phase. Understanding the risks and setting realistic expectations can help you make an informed decision about undergoing breast reduction surgery. While changes in sensation are a consideration, many patients find that the benefits of breast reduction, such as reduced back and shoulder pain and improved body contour, outweigh these risks. However, each person's values and circumstances are unique, so personal priorities will significantly influence this decision.
Q13. Should I lose weight before getting a breast reduction surgery?	Deciding whether to lose weight before undergoing breast reduction surgery (BRS) is an important consideration and should be made based on individual circumstances. Here are key points to consider that can help you make an informed decision: Impact on Surgical Outcomes Stability of Results: Weight fluctuations can significantly impact the size and shape of your breasts. If you lose weight after the surgery, your breasts could reduce further, possibly altering the aesthetic results achieved from the reduction. Conversely, gaining weight could lead to an increase in breast size again. Achieving Proportionate Results: Surgeons aim to create a breast size that is proportionate to your body frame. If you are planning to lose a significant amount of weight, doing so before the surgery can help achieve a more balanced and desired outcome. Health Considerations Reduced Surgical Risks: Being closer to your ideal weight can decrease the risk of complications during and after surgery. Obesity and high body mass index (BMI) are associated with higher risks of surgical complications such as infection, poor wound healing, and adverse reactions to anesthesia. Overall Recovery: Weight loss and being in good physical health can also contribute to a smoother and faster recovery process. Psychological and Physical Readiness Body Image and Satisfaction: Weight loss before surgery might change your perspective on how much breast tissue you actually want removed. What might seem like a desirable size when you are heavier could change once you reach a lighter weight. Long-term Satisfaction: Achieving or being close to your goal weight prior to surgery can contribute to longer-term satisfaction with the results, as the size and shape achieved during surgery are more likely to remain stable. Practical Considerations Time and Effort: Weight loss requires time and effort. It’s important to approach weight loss in a healthy and sustainable manner, ideally under the guidance of healthcare professionals. Consider whether you have the time and resources to commit to weight loss before planning your surgery. Consultation with Your Surgeon Professional Guidance: It’s crucial to discuss your current weight and any weight loss plans with your plastic surgeon during the consultation. The surgeon can provide guidance based on your specific body type, the amount of breast tissue, and your overall health. If losing weight is a goal for you, ideally, this should be achieved before breast reduction surgery to optimize both the safety and the aesthetic outcomes of the procedure. However, each case is unique, so professional advice from your healthcare provider and plastic surgeon is essential to make the best decision tailored to your personal needs and circumstances.
Q14. Should I get a breast reduction surgery before or after having kids?	Deciding whether to have breast reduction surgery before or after having children involves considering several factors related to personal goals, the timing of family planning, and the potential impact on breastfeeding. Here’s a detailed look at the considerations: Breastfeeding Considerations Potential Impact on Ability to Breastfeed: Breast reduction surgery can sometimes affect your ability to breastfeed, depending on the technique used. Some methods preserve more of the milk ducts connected to the nipples than others. Discussing these options with your surgeon can help you understand the potential risks and make an informed decision based on your desire to breastfeed in the future. Changes in Breast Tissue During Pregnancy Breast Changes During Pregnancy and Postpartum: Pregnancy and breastfeeding can lead to significant changes in the size and shape of your breasts. These changes might alter the results achieved from a previous breast reduction surgery. There's a possibility that you might desire or require additional procedures to address these changes after childbirth and breastfeeding. Timing and Family Planning Consider Your Family Planning Timeline: If you plan to have children in the near future, it may be worth waiting until after you've finished building your family before undergoing breast reduction. This strategy can help ensure that the results of the surgery are not compromised by the breast changes associated with pregnancy. Symptom Severity Addressing Immediate Discomfort: If your large breast size is causing significant physical discomfort, such as back pain, neck pain, or skin issues, and these symptoms are severely impacting your quality of life, it might be beneficial to proceed with the surgery before having children. Alleviating these symptoms can also make the experience of pregnancy more comfortable. Psychological and Physical Readiness Emotional Well-being: Consider your emotional state and overall readiness. Breast reduction surgery can have a significant positive impact on self-esteem and body image for many women, which might be an important factor in your decision-making process. Consultation with a Specialist Professional Advice: A consultation with a plastic surgeon can provide valuable insights. The surgeon can assess the physical considerations of your breasts, discuss how pregnancy might alter your results, and help you weigh the pros and cons based on your specific circumstances. In summary, the decision to undergo breast reduction surgery before or after having children is highly personal and should be based on your immediate needs, family planning goals, and potential desire to breastfeed. Engaging in a thorough discussion with a qualified plastic surgeon can help ensure that you make the best decision for your long-term satisfaction and health.
Q15. What are the risks of BRS?	Breast reduction surgery (BRS), like any surgical procedure, carries certain risks and potential complications. While many patients experience significant benefits and improvements in quality of life after surgery, it's important to be aware of the possible adverse effects when considering this option. Here are some of the common risks associated with breast reduction surgery: 1. Anesthesia Risks Like all procedures requiring anesthesia, there is a risk of adverse reactions, which can include respiratory or cardiac malfunction during or after surgery. 2. Bleeding There is a risk of bleeding during or after the surgery, which might require a return to surgery to control and drain the blood. 3. Infection The surgical wounds can become infected, which may require treatment with antibiotics and sometimes additional surgery. 4. Poor Wound Healing The incisions from breast reduction surgery may heal poorly or take longer than expected. This can result in conspicuous scarring, skin loss, or other aesthetic issues. 5. Changes in Nipple or Breast Sensation Temporary or permanent changes in nipple or breast sensation may occur after breast reduction surgery. While many patients regain sensation, some may experience permanent numbness. 6. Scarring Scarring is inevitable after breast reduction surgery, though the severity varies. Scars typically improve over time but may be more prominent in some individuals. 7. Asymmetry Breasts may not be perfectly symmetrical in size or shape after surgery, and nipple position may vary between the two breasts. Further surgery might be required to correct significant asymmetry. 8. Difficulty Breastfeeding Depending on the technique used, some women may find it challenging to breast
